# Adaptive MANET Multipath Routing Algorithm Based on the Simulated Annealing Approach

**DOI:** 10.1155/2014/872526

**Published:** 2014-06-16

**Authors:** Sungwook Kim

**Affiliations:** Department of Computer Science, Sogang University, Sinsu dong 1, Mapo-ku, Seoul 121-742, Republic of Korea

## Abstract

Mobile ad hoc network represents a system of wireless mobile nodes that can freely and dynamically self-organize network topologies without any preexisting communication infrastructure. Due to characteristics like temporary topology and absence of centralized authority, routing is one of the major issues in ad hoc networks. In this paper, a new multipath routing scheme is proposed by employing simulated annealing approach. The proposed metaheuristic approach can achieve greater and reciprocal advantages in a hostile dynamic real world network situation. Therefore, the proposed routing scheme is a powerful method for finding an effective solution into the conflict mobile ad hoc network routing problem. Simulation results indicate that the proposed paradigm adapts best to the variation of dynamic network situations. The average remaining energy, network throughput, packet loss probability, and traffic load distribution are improved by about 10%, 10%, 5%, and 10%, respectively, more than the existing schemes.

## 1. Introduction

Due to the explosive growth of wireless communication technology, mobile ad hoc networks (MANETs) have been used in many practical applications in the commercial, military, and private sectors. MANETs are self-creating, self-organizing, and autonomous systems of mobile hosts connected by wireless links with no static infrastructure such as base station. When making such networks operational, a key question is how to effectively decide routing paths, given the dynamic nature of the system and the limited knowledge of the network topology. In recent times, a lot of attention has been attracted to designing efficient routing protocols for efficient MANET operations [1–4].

During the operation of MANETs, unexpected growth of traffic may develop in a specific routing path; it may create local traffic congestion. In order to alleviate this kind of traffic overload condition, load balancing strategy should be employed. In MANETs, the meaning of load balancing is to ease out the heavy traffic load in a specific path, which can ensure the balanced network resource assumption. To ensure the load balancing, multipath routing algorithms have been developed. Multipath routing algorithm establishes multiple paths between a source and a destination node and spreads the traffic load along multiple routes. It can alleviate traffic congestion in a specific path. Therefore, multipath routing algorithms can provide the route resilience while ensuring the reliability of data transmission [5, 6].

Nowadays, metaheuristic approach is widely recognized as a practical perspective to be implemented for real world network operations [7–9]. Traditionally, metaheuristic algorithms try to improve a candidate solution iteratively with regard to a given measure of quality. Even though this approach does not guarantee an optimal solution, it can be widely applied to various network control problems. Simulated annealing is a well-known probabilistic metaheuristic algorithm for finding an effective solution [10–12]. To adaptively make a routing decision, the basic concept of simulated annealing approach can be adopted.

Motivated by the facts presented in the above discussion, a new multipath routing scheme is proposed based on the simulated annealing approach. In this work, we do not focus on trying to get an optimal solution itself, but, instead, an adaptive online feedback model is adopted. Therefore, the proposed scheme repeatedly estimates the current network situations and dynamically makes a control decision. This approach can significantly reduce the computational complexity and overheads. Due to this reason, wireless nodes are assumed to be self-interested agents and make local decisions in a distributed manner. Therefore, routing packets are adaptively distributed through multiple paths in pursuit of the main goals such as load balancing and network reliability. Under diverse network environment changes, the proposed scheme tries to approximate an optimal network performance. The important features of our proposed scheme are (i) interactive process to get an efficient network performance, (ii) distributed approach for large-scale network operations, (iii) dynamic adaptability to current network, and (iv) feasibility for practical implementation.

### 1.1. Related Work

Recently, several routing schemes for ad hoc networks have been presented in research literature. Leu et al. developed the multicast power greedy clustering (MPGC) scheme [13], which is an adaptive power-aware and on-demand multicasting algorithm. The MPGC scheme uses greedy heuristic clustering, power-aware multicasting, and clustering maintenance techniques that maximize energy efficiency and prolong network lifetime.

To improve the network reliability and reduce the network traffic, Kim et al. propose the double-layered effective routing (DLER) scheme for peer-to-peer network systems [14]. This scheme first chooses the shortest routing paths among possible routing paths and selects the path associated with the relay peer who has lower mobility to improve the reliability of the system. Therefore, in the DLER scheme, the lower mobility of relay peers contributes to both the stability of clusters and the robustness of the system.

Hieu and Hong proposed the entropy-based multirate routing (EMRR) scheme [15]. This scheme introduces a new approach to modeling relative distance among nodes under a variety of communication rates, due to node's mobility in MANETs. When mobile nodes move to another location, the relative distance between communicating nodes will directly affect the data rate of transmission. Therefore, the stability of a route is related to connection entropy. Taking into account these issues, the link weight and route stability based on connection entropy are considered as a new routing metric. In the EMRR scheme, the problem of determining the best route is formulated as the minimization of an object function formed as a linear combination of the link weight and the connection uncertainty of that link.

The ant-colony based routing algorithm (ARA) scheme was proposed [16]; in this scheme, swarm intelligence and ant-colony metaheuristic techniques are used. This scheme consists of three phases: route discovery, route maintenance, and route failure handling. In the route discovery phase, new routes between nodes are discovered using forward and backward ants. Routes are maintained by subsequent data packets; that is, as the data traverse the network, node pheromone values are modified to reinforce the routes.

Wang et al. developed the logical hypercube-based virtual dynamic backbone (HVDB) scheme for an *n*-dimensional hypercube in a large-scale MANET [17]. The HVDB scheme is a proactive, QoS-aware, and hybrid multicast routing protocol. Owing to the regularity, symmetry properties, and small diameter of the hypercube, every node plays almost the same role. In addition, no single node is more loaded than any other node, and bottlenecks do not exist, unlike the case in tree-based architectures. In particular, the HVDB scheme can satisfy the new QoS requirements—high availability and good load balancing—by using location information.

Barolli et al. proposed the genetic algorithm based routing (GAR) scheme for mobile ad hoc networks [18]. In the GAR scheme, only a small number of nodes are involved in route computation because a small population size is used. As a result, routing information is transmitted only for the nodes present in that population. Different routes are ranked by sorting; the first route is the best one, and the remaining routes are used as backup routes. Because a tree-based genetic algorithm method is used in the GAR scheme, the delay and transmission success rate are considered as QoS parameters in this scheme.

The incentive-based repeated routing (IRR) scheme in [19] is an incentive-based routing model that captures the notion of repetition. To provide a desirable solution, the IRR scheme examines certain fundamental properties to govern the behavior of autonomous agents. The distributed routing mechanism (DRM) scheme in [20] is an adaptive and scalable routing scheme for wireless ad hoc networks. This scheme provides a cost-efficient routing mechanism for strategic agents. In addition, the DRM scheme is designed to maximize the benefit of each agent.

The proactive congestion reduction (PCR) scheme in [5] focuses on adaptive routing strategies to help congestion reduction. Based on a nonlinear optimization method for multipath routings, the PCR scheme calculates a traffic splitting vector that determines a near-optimal traffic distribution over routing paths. The shortest multipath source (SMS) scheme [6] is one of the most generally accepted on-demand dynamic routing schemes that build multiple shortest partial disjoint paths. The SMS scheme uses node-disjoint secondary paths to exploit fault tolerance, load balancing, and bandwidth aggregation. All the earlier work has attracted a lot of attention and introduced unique challenges. However, these existing schemes have several shortcomings as described in Section 3. Compared to the PCR scheme and the SMS scheme [5, 6], the proposed scheme attains better performance for wireless network managements.

This paper is organized as follows.  Section 2 describes the proposed algorithms in detail. In Section 3, performance evaluation results are presented along with comparisons with the schemes proposed in [5, 6]. Finally, in Section 4, concluding remarks are given and some directions are identified for future work.

## 2. Proposed MANET Routing Algorithms

Multipath routing algorithms are designed to split and transmit the traffic load through two or more different paths to a destination simultaneously. In this paper, we propose a new multipath routing scheme to balance the network load while ensuring efficient network performance.

### 2.1. Path Setup Algorithm

Usually, wireless link capacity continually varies because of the impacts from transmission power, interference, and so forth. Therefore, it is important to estimate the current link status by considering several control factors. To configure the adaptive multihop routing path, the proposed algorithm defines a link cost (*L*_*P*) for each link to estimate the degree of communication adaptability [21, 22]. In order to relatively handle dynamic network conditions, the *L*_*P* value from the node *i* to the node *j* is obtained as

(1) L_Pij=[(1−α)×Cij+α×(1−Θj(t))]+[ω×(1−Ψij(t))],s.t.,  α=EiEM,  Cij=dijDM,  Ψij(t)=κtij(κtij+ϑtij),

where *d*_*ij*_ is the distance from the node *i* to the node *j* and *E*_*i*_ is the remaining energy of the node *i*. *E*_*M*_ and *D*_*M*_ are the initial energy and the maximum coverage range of each node. Therefore, the *d*_*ij*_ and *E*_*i*_ are normalized by the *D*_*M*_ and *E*_*M*_; the range is varied from 0 to 1. Θ_*j*_(*t*) is the entropy for the node *j* at the time (*t*). Usually, entropy is the uncertainty and a measure of the disorder in a system. It represents the topological change, which is a natural quantification of the effect of node mobility on MANET's connectivity service [23]. In this work, the basic concept of entropy is adopted for supporting and evaluating stable routing routes. For the mobile node *j*, the entropy Θ_*j*_(*t*) is calculated as follows [23]:

(2) Θj(t)=−∑k∈FjPk(t,Δt)×log⁡Pk(t,Δt)log⁡C(Fj),s.t.,  Pk(t,Δt)=aj,k∑i∈Fjaj,i,

where Δ_*t*_ is a time interval. *F*_*j*_ denotes the set of the neighboring nodes of node *j*, and *C*(*F*_*j*_) is the cardinality (degree) of set *F*_*j*_. To estimate the stability of a part of a specific route, *a*_*j*,*i*_ represents a measure of the relative mobility among two nodes *j* and *i* as

(3) aj,i=1I_T×∑l=1I_T|v(j,i,tl)|,s.t.,  v(j,i,t)=v(j,t)−v(i,t),

where *v*(*j*, *t*) and *v*(*i*, *t*) are the velocity vectors of node *j* and node *i* at time *t*, respectively. *I*_*T* is the number of discrete times *t*_*l*_ that mobility information can be calculated and disseminated to other neighboring nodes within time interval Δ_*t*_. *v*(*j*, *i*, *t*) is the relative velocity between nodes *j* and *i* at time *t*. Any change can be described as a change of variable values *a*_*j*,*i*_ in the course of time *t* such as *a*_*j*,*i*_(*t*) → *a*_*j*,*i*_(*t* + Δ_*t*_). The entropy Θ_*j*_(*t*) is normalized as 0 ≤ Θ_*j*_(*t*) ≤ 1. If Θ_*j*_(*t*) value is close to 1, the part of the route that represents the links of the path associated with an intermediate node *j* is stable. If Θ_*j*_(*t*) value is close to 0, the local route is unstable [23]. In (1), Ψ_*ij*_(*t*) is the link *ij*'s trust value at the time *t*. After the *t*th iteration, Ψ_*ij*_(*t*) is using the number of packets successfully serviced in the link *ij* (*κ*_*t*_^*ij*^) divided by the total number of packets that have been sent from the node *i* to the relay node *j* (*κ*_*t*_^*ij*^ + *ϑ*_*t*_^*ij*^).

To relatively estimate the current link situation by using (1), the control parameters *α* and *ω* should be adjusted dynamically. The *C*_*ij*_ reflects the cost of the wireless communication; the closer a next node, the more attractive for routing due to the less communication cost. The *E*_*i*_ is the current residual energy of node *i*, which reflects the remaining lifetime of a wireless node. Due to the characteristics of wireless propagation, the energy consumption rate for wireless communications is strongly related to the internode distance. The parameter *α* controls the relative weights given to distance and entropy of corresponding relay node. Under diverse network environments, a fixed value of *α* cannot effectively adapt to the changing conditions [21, 22]. In this paper, we treat it as an online decision problem and adaptively modify *α* value. When the remaining energy of the node *i* is high, we can put more emphasis on the stability status of next node *j*, that is, on (1 − Θ_*j*_(*t*)). In this case, a higher value of *α* is more suitable. When the remaining energy of the node *i* is not enough due to traffic overhead, the path selection should strongly depend on the energy dissipation for data transmission. In this case, a lower value of *α* is more suitable for the energy consumption rate, that is, on *C*_*ij*_, since the distance between two neighbor nodes directly affects the energy consumption rate. In the proposed algorithm, the value of *α* of the node *i* is dynamically adjusted based on the current rate of its remaining energy per initially assigned energy (*E*_*i*_/*E*_*M*_). Therefore, the system can be more responsive to current network conditions by the real-time network monitoring. The parameter *ω* is an impact factor to evaluate the trust level of the link. In this paper, to avoid the detrimental packet loss effect, each link's trust level is fully considered to estimate *L*_*P* value; the *ω* value is fixed as 1.

The *L*_*P* value can represent the normalized communication cost of each link. With the *L*_*P* value, we define the path cost parameter (PC) to calculate total routing path cost; PC is computed as the sum of all link costs from the source node to the current node. Based on the PC value, the proposed routing algorithm constructs adaptive multihop routing paths to reach the destination node. At the initial time for routing operations, the source node broadcasts its initial PC value (i.e., PC = 0). Within the power coverage area, message receiving relay nodes individually estimate the link cost according to (1) and estimate its PC value as PC + *L*_*P*. Some nodes can receive multiple PC values from reachable different neighbor nodes. For self-organizing and independent-effective controlling, each node keeps this information. For example, the node *i* can have received multiple PC values, that is, PC_1_, PC_*k*_, and PC_*N*_*i*__, where PC_*k*_ is the receiving PC value of the message-sending neighbor node *k* (1 ≤ *k* ≤ *N*_*i*_) and *N*_*i*_ is the number of total reachable neighbor nodes. In this case, the node *i* calculates its own PC_*i*_ value as follows:

(4) PCi=argmin⁡k∈Ni(PCk+L_Pik).

According to (4), the node *i* adaptively selects one neighbor node as a relay node while minimizing PC_*i*_ value, which potentially incorporates more global network information. The estimated PC value is recursively forwarded to establish the routing path. This route formation process is repeated until all available multipaths from the source to the destination node are configured.

### 2.2. Simulated Annealing Routing Algorithm

Generally, multipath routing algorithms face an essential challenge—how to distribute the volume of traffic to a specific path. In order to produce good solutions within a reasonable amount of computer time, the proposed scheme does not seek the optimal allocation. Based on feedbacks of the real-time traffic measurements, it is designed in a simple but efficient metaheuristic algorithm.

Simulated annealing (SA) is a well-known metaheuristic method that has been applied successfully to combinatorial optimization problems [8]. The term simulated annealing derives from the roughly analogous natural phenomena of annealing of solids, which is accomplished by heating up a solid and allowing it to cool down slowly so that thermal equilibrium is maintained. Each step of the SA process replaces the current solution by a random “nearby” solution, chosen with a probability that depends on the difference between the corresponding function values and on a global parameter *T* (called the temperature). The *T* is gradually decreased during the process to reach steady state or thermal equilibrium [8, 10, 12].

In the proposed algorithm, the SA approach is used to solve the multipath routing problem. The basic concept of the proposed algorithm is to proportionally load traffic on each route according to its adaptability. To transmit packets, each node selects a next relay node based on the PC information. From the point of view of the node *i*, selection probability of the neighbor node *k* (SP_*k*_) is defined as follows:

(5) SPk=TCk∑j=1nTCj, where  TCk=1−(PCj+L_Pij)∑j=1n(PCj+L_Pij),  k∈Ni,

where *n* is the total number of neighbor nodes. Based on the* roulette-wheel* function [24] of SP values, a next relay node is temporarily selected. For example, the probability of node *k*'s selection is SP_*k*_. Therefore, we can make the more adaptable nodes more likely to be selected than the less adaptable nodes. In addition, to avoid a local optimal solution, the Boltzmann probability (BP) is adopted. The BP is defined as follows [8]:

(6) BP=exp⁡(−Npc−CpcT(t)),

where *N*_pc_ is the SP value of new selected node and *C*_pc_ is the SP value of previously connected node. In (6), the difference between *N*_pc_ and *C*_pc_ (i.e., *N*_pc_ − *C*_pc_) means the path adaptability alteration. *T*(*t*) is a parameter to control the BP value. Metaphorically, it is the time *t*'s* temperature* of the system. As an annealing process, the *T*(*t*) is decreased according to a cooling schedule. At the beginning of the annealing algorithm run, the initialization temperature is high enough so that possibility of accepting any decision changes whether it improves the solution or not. While time is ticking away, the *T*(*t*) value decreases until the stopping condition is met. In this paper, *T*(*t*) value is set to the current ratio of the remaining packet amount to the total routing packet amount.

At the routing decision time, there are two cases.

- If the *N*_pc_ value is higher than the *C*_pc_ (i.e., *N*_pc_ − *C*_pc_ ≥ 0), the new selected neighbor node replaces the current relay node.
- If the *N*_pc_ value is less than the *C*_pc_ value (i.e., *N*_pc_ − *C*_pc_ < 0), the new selected neighbor node is not eligible to replace the current relay node. However, this node might still be accepted as a new relay node to potentially avoid local optima. It is analogous to the uphill move acceptance to reach an optimal point. In this case, a random number *X* is generated, where *X* is in the range of {0 ⋯ 1}.
  - If the *X* is less than BP (i.e., *X* < BP), the new selected neighbor node replaces the current relay node.
  - Otherwise, the current routing route is not changed.

Based on the SA approach, individual nodes in our proposed scheme locally make routing decisions to select next relay nodes. In an entirely distributed fashion, this hop-by-hop path selection procedure is recursively repeated until the packet reaches the destination node. Therefore, our proposed routing algorithm can have the self-adaptability for network dynamics.

### 2.3. The Main Steps of MANET Routing Algorithm

In this paper, we propose a new multipath routing algorithm for wireless mobile ad hoc networks. In the proposed scheme, routing is guided by employing a simulated annealing process. Therefore, self-interested ad hoc nodes make routing decisions according to private preferences while adapting the current network situations. To solve the dynamic and distributed routing problem, the main steps of the proposed multipath routing algorithm are given next.

**Step 1 .**

Each node dynamically estimates the *d*, *C*, *E*, Θ(·), Ψ(·), and *α* values based on the real-time measurement.

**Step 2 .**

The *L*_*P* value is locally calculated according to (1).

**Step 3 .**

At the initial time for routing operations, the source node broadcasts the initial PC value to neighbor nodes. Each node calculates its PC value by using (4) and recursively forwards this information.

**Step 4 .**

Based on the PC value, route configuration process continues repeatedly until all available multipaths from the source to the destination node are configured.

**Step 5 .**

To transmit packets, each relay node temporarily selects a next relay node with the selection probability, which is estimated according to (5).

**Step 6 .**

If the *N*_pc_ value is higher than the *C*_pc_ (i.e., *N*_pc_ − *C*_pc_ > 0), the new selected neighbor node replaces the current relay node; proceed to Step 8. Otherwise, go to Step 7.

**Step 7 .**

When the *N*_pc_ value is less than the *C*_pc_ value (i.e., *N*_pc_ − *C*_pc_ < 0), a random number *X* is generated. If a generated *X* is less than the BP (i.e., *X* < BP), the new selected neighbor node replaces the current relay node. Otherwise, the established routing route is not changed.

**Step 8 .**

In an entirely distributed fashion, this hop-by-hop path selection procedure is recursively repeated until the packet reaches the destination node.

## 3. Performance Evaluation

In this section, the effectiveness of the proposed algorithms is validated through simulation; we propose a simulation model for the performance evaluation. With a simulation study, the performance superiority of the proposed multipath routing scheme can be confirmed. The assumptions implemented in our simulation model were as follows.

- 100 nodes are distributed randomly over an area of 500 × 500 meter square.
- Each data message is considered CBR traffic with the fixed packet size.
- Network performance measures obtained on the basis of 50 simulation runs are plotted as functions of the packet generation per second (packets/s).
- Data packets are generated at the source according to the rate *λ* (packets/s), and the range of offered load was varied from 0 to 3.0.
- The bandwidth of the wireless link was set to 5 Mb/s and the* unit_time* is one second.
- The source and destination nodes are randomly selected.
- For simplicity, we assume the absence of noise or physical obstacles in our experiments.
- The mobility of each mobile node is randomly selected from the range of 0–10 m/s, and mobility model is random way point model.
- At the beginning of simulation, all nodes started with an initial energy of 10 joules.
- Three different traffic types were assumed; they were generated with equal probability.

Table 1 shows the traffic types and system parameters used in the simulation. Each type of traffic has its own requirements in terms of bandwidth and service time. In order to emulate a real wireless network and for a fair comparison, we used the system parameters for a realistic simulation model [21, 22].

**Figure 1:**
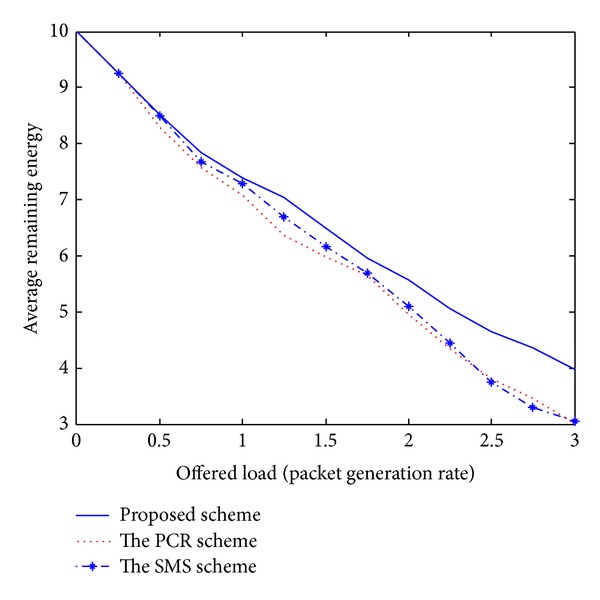
Average remaining energy.

**Figure 2:**
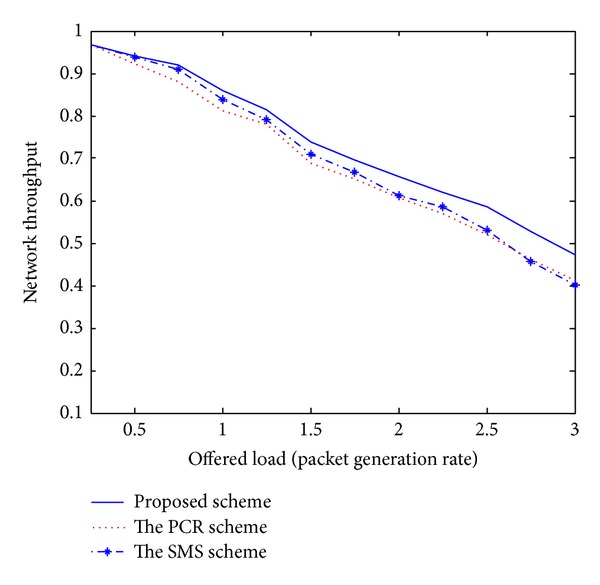
Network throughput.

**Figure 3:**
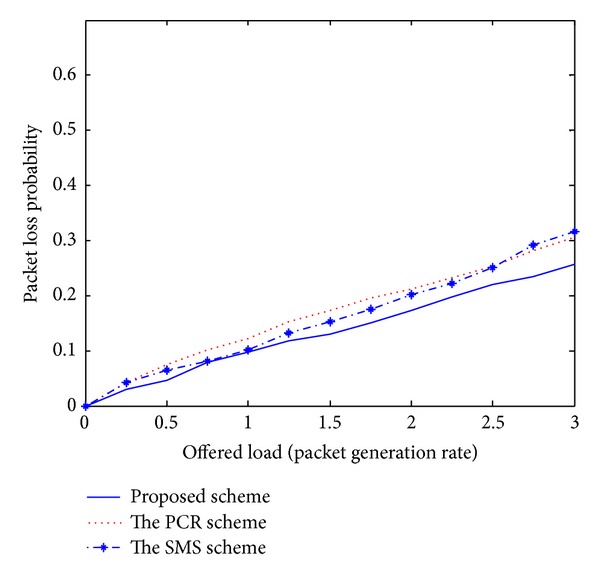
Packet loss probability.

**Figure 4:**
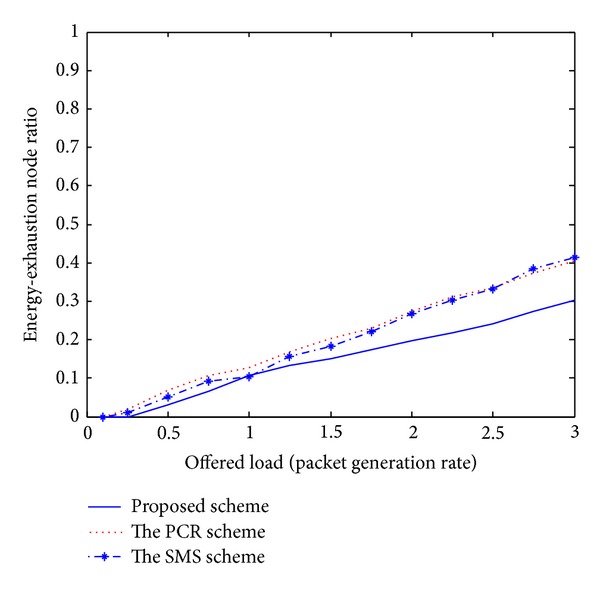
Energy-exhaustion ratio.

**Figure 5:**
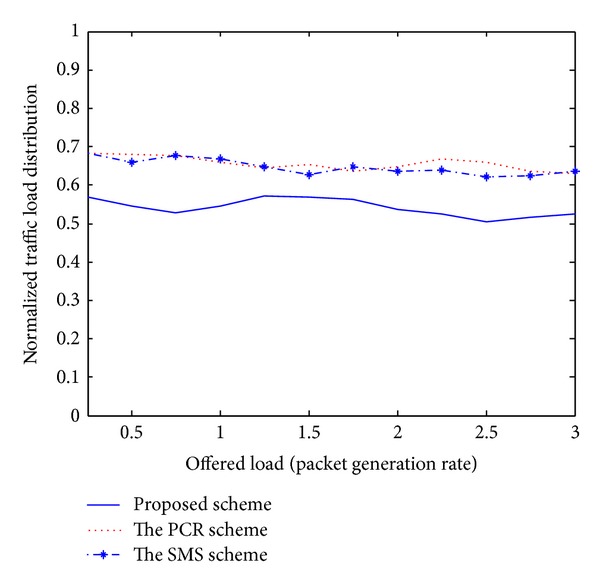
Normalized traffic load distribution.

Recently, the PCR scheme [5] and the SMS scheme [6] have been published and introduced unique challenges for the issue of multipath routing in MANETs. Even though these existing schemes have presented novel multipath routing algorithms, there are several disadvantages. First, these schemes cannot adaptively estimate the current network conditions. Therefore, each node is unaware of effective routing paths to reach a destination. Second, some nodes carry a disproportionately large amount of the entire traffic, drastically decreasing the throughput of the flows they forward. Third, the PCR and SMS schemes are based on a centralized approach. The ideas for practical implementations are left for future study. As mentioned earlier, we compare the performance of the proposed scheme with these existing schemes to confirm the superiority of the proposed approach. In our simulation analysis of Figures 1–5, the *x*-axis (a horizontal line) marks the traffic intensities, which is varied from 0 to 3.0. The *y*-axis (a vertical line) represents the normalized value for each performance criterion.

Figure 1 compares the performance of each scheme in terms of the average remaining energy of wireless nodes. To maximize a network lifetime, the remaining energy is an important performance metric. All the schemes have similar trends. However, based on (1), the proposed scheme effectively selects the next routing link by considering the remaining energy information. Therefore, we attain much remaining energy under heavy traffic load intensities; it guarantees a longer node lifetime.

Figure 2 shows the performance comparison of network throughput. Usually, network throughput is the rate of successful message delivery over a communication channel. The throughput is usually measured in bits per second (bit/s or bps) and sometimes in data packets per second or data packets per time slot. In this work, network throughput is defined as the ratio of data amount received at the destination nodes to the total generated data amount. For a fair comparison, it is the best realistic way. Due to the inclusion of the adaptive online approach, the proposed scheme can have the best throughput gain.

In Figure 3, the packet loss probabilities are presented; packet loss means the failure of one or more transmitted packets to arrive at their destinations. As the offered traffic load increases, wireless nodes will run out of the energy or capacity for data transmissions and data packets are likely to be dropped. Therefore, the packet loss probability increases linearly with the traffic load. Based on the real-time online manner, our dynamic SA approach can improve the system reliability, so we achieve a lower packet loss rate than other schemes under various traffic loads.

The curves in Figures 4 and 5 indicate the average energy-exhaustion ratio and normalized traffic load distribution. In this paper, traffic load distribution means the average rate of traffic dispersion among wireless nodes. In an entirely distributed fashion, individual node in our scheme monitors the current network situation and updates all control parameters periodically for the adaptive routing. Therefore, under various system constraints, the proposed scheme is able to decrease the number of energy expiration nodes and adaptively distribute routing packets to avoid traffic congestions, which is highly desirable property for the MANET management.

The simulation results shown in Figures 1–5 demonstrate that the proposed multipath routing scheme generally exhibits better performance compared with the other existing schemes [5, 6]. Based on the adaptive simulated annealing approach, the proposed scheme constantly monitors the current traffic conditions and gets an efficient solution. Through the simulation experiments, it could be seen that the proposed strategy is proved to be an effective paradigm to solve complex routing problems in a dynamic network environment.

## 4. Summary and Conclusions

Recent advances in wireless technology and availability of mobile computing devices have generated a lot of interest in mobile ad hoc networks. For these networks, the biggest challenge is to find routing paths to satisfy varying requirements. In this paper, new multipath routing algorithms are developed based on the effective simulated annealing approach. For real network implementation, the proposed scheme is designed in self-organizing, dynamic online, and interactive process. Therefore, each individual node has an ability to provide more adaptive control mechanism and makes a local routing decision to find an efficient path. Under dynamic network environments, this approach can dynamically reconfigure the established path to adapt to network changes. From simulation results, the proposed scheme outperforms existing schemes in terms of network reliability, energy efficiency, and so forth.

In the future, we expect our methodology to be useful in developing new adaptive ad hoc routing algorithms. In particular, the metaheuristic approach can be extended to support delay sensitive data services. In addition, the basic concept of adaptive online algorithms has become an interesting research topic in highly mobile ad hoc networks.

## Figures and Tables

**(a):** 

Traffic type	Bandwidth requirement	Connection duration (ave./sec)
I	128 Kbps	60 sec (1 min)
II	256 Kbps	120 sec (2 min)
III	512 Kbps	180 sec (3 min)

**(b):** 

Parameter	Value	Description

*unit_time *	1 second	Equal interval of time axis
*e*_dis_	1 pJ/bit/m^2^	Energy dissipation coefficient for the packet transmission
*E*_co⁡_	10 nJ/bit	System parameter for the electronic digital coding energy dissipation
*D*_*M*_	10 m	Maximum wireless coverage range of each node
*E*_*M*_	10 joules	Initial assigned energy amount of each node
*ω*	1	The weighted factor for the trust level
*I*_*T*	10 seconds	The number of discrete times to estimate entropy
*X*	0~1	Generated random number

**(c):** 

Parameter	Initial	Description	Values

*α*	1	The ratio of remaining to initial energy of node	0~1 (*E*_*i*_/*E*_*M*_)
*T*(*t*)	1	The ratio of remaining to initial packet amount at time *t *	0~1
